# Lifestyle interventions in comorbid mental and physical illness: A systematic review protocol

**DOI:** 10.4102/sajp.v79i1.1848

**Published:** 2023-04-21

**Authors:** Sandy Lord, Vaneshveri Naidoo, Karien Mostert

**Affiliations:** 1 Department of Physiotherapy, Faculty of Health Sciences, University of the Witwatersrand, Johannesburg, South Africa; 2 Department of Physiotherapy, Faculty of Health Sciences, University of Pretoria, Johannesburg, South Africa

**Keywords:** lifestyle interventions, mental health, physical health, therapy, comorbidities, pain

## Abstract

**Background:**

Patients with mental health disorders (MHDs) often present with chronic illness and complain of pain and poor physical health. They present with a high burden of disease and poor quality of life. Significant associations between MHDs and chronic illness have been found. Lifestyle interventions are cost-effective strategies, which seem to be effective in managing comorbid mental and physical health disorders. Therefore, a summary of the evidence and clinical practice guidelines is needed in South Africa.

**Objectives:**

Our study will aim to determine the effectiveness of lifestyle interventions on health-related quality of life, in patients with comorbid mental and physical health disorders.

**Method:**

The systematic review will be conducted using the Joanna Briggs Institute (JBI) methodology for systematic reviews of effectiveness. MEDLINE (Ovid), CINAHL (EBSCO), LiLACS, Scopus, Physiotherapy Evidence Data Base (PEDro) and Cochrane Central Register of Controlled Trials will be searched. A three-step search strategy will identify published literature in all languages from 2011 to 2022. Critical appraisal of all included studies will be performed, and the relevant data will then be extracted. Where possible, data will be pooled in a statistical meta-analysis.

**Results:**

The results will provide the best available evidence regarding lifestyle interventions in the management of patients with comorbid mental and physical health disorders.

**Conclusion:**

Our review will provide evidence on the effectiveness of lifestyle interventions in the management of patients with comorbid mental and physical health disorders.

**Clinical implications:**

The results may assist in determining the best use of lifestyle interventions in the management of patients with MHDs and comorbidities.

## Introduction

Mental health disorders (MHDs) affect many bodily systems, which in turn can affect health and well-being and have been associated with increased disability in work, family and social environments (World Health Organization [WHO] 2022a, 2022b). Mental health disorders include anxiety, mood disorders and neurodevelopmental disorders (WHO 2022b). Substance use disorders often co-occur with MHDs and either contribute to the MHD or are a cause thereof. Patients with MHDs often complain of pain and poor physical health. Epidemiological studies have found significant associations between MHDs and non-communicable diseases (NCDs) (Ee et al. 2020; Stein et al. 2019). The evidence indicates that patients with severe MHDs have higher rates of cancers, respiratory disorders, cardiovascular disease, obesity and diabetes (De Hert et al. 2011; Ee et al. 2020; Stein et al. 2019). People with MHDs have a lower life expectancy than the general population. The main cause of mortality is chronic illness because of poor lifestyle factors (De Hert et al. 2011; Hodgson, Mcculloch & Fox 2011; Lee et al. 2017; Manger 2019). The side effects of psychotropic medication include metabolic syndrome, weight gain, neurological disorders, an increased risk of diabetes and coronary artery disease and therefore are a cause of many physical comorbidities (Ee et al. 2020). Pain and depression also often co-exist, with more than half of patients with MHDs experiencing pain (Goesling, Clauw & Hassett 2013). Mental and physical health disorders are not only highly comorbid but are also mutually reinforcing (Opie et al. 2021). As a result, patients with pain, chronic illness and comorbid psychiatric conditions have a high burden of disease and poorer quality of life (QoL) (Chen et al. 2006).

Quality of life is an important indicator of health (Owczarek 2010). It is the degree to which a healthy person participates in activities of daily living (Encylopedia Britannica 2022; Merriam-Webster 2023). According to the WHO (2012), QoL is:

[*A*]n individual’s perception of their position in life in the context of the culture and value systems in which they live and in relation to their goals, expectations, standards and concerns. (p. 1)

Quality of life is multidimensional and has four domains, which include mental and physical health as well as social and functional health (Aaronson 1988; Owczarek 2010).

Patients often have multiple unhealthy lifestyle factors, which may in turn worsen their physical and mental health (Zaman, Hankir & Jemni 2019). Poor mental health often leads to social isolation, lower motivation and engagement and less social or community involvement. These patients are, therefore, often less likely to implement lifestyle changes (Ee et al. 2020). Risk factors for chronic NCDs commonly cluster in people with MHDs (Stein et al. 2019). The four main lifestyle factors (poor diet, lower levels of physical activity, smoking and excessive alcohol consumption) often occur together. Patients with unhealthy eating habits often have lower levels of physical activity, both of which can contribute to obesity and cardiovascular disease. Smoking may cause chronic respiratory conditions and as such lead to decreased physical activity (Zaman et al. 2019). Many of the lifestyle factors, which contribute to these disorders, are modifiable and interventions that address these lifestyle factors should be included in the management of persons with MHDs and NCDs (Lee et al. 2017; Probst & Skjaerven 2017).

Lifestyle interventions include sleep, physical activity and exercise, diet, stress management and substance cessation (Ford et al. 2009). They are indicated for people with MHDs as an alternative or adjunct to usual care (Manger 2019). Lifestyle interventions are cost-effective strategies that could help prevent diabetes and heart attacks (Manger 2019). Exercise, mindfulness-based interventions and a healthy diet are also effective in the management of both mental and physical health disorders. Exercise, in particular, provides symptomatic improvements when used as an adjunctive therapy in major depressive disorders and may be used as a first-line therapy for mild to moderate depression. Furthermore, exercise reduces the risk of cancers, cardiovascular disease and diabetes, which is often associated with increased sedentary behaviour (Ee et al. 2020).

Lifestyle interventions are cost-effective therapies aimed at behaviour modification and can be delivered by diverse members of the inter-professional team. Medication and psychotherapy are usually the first line of treatment. Lifestyle interventions have been shown to be effective as adjunctive therapies for mental health. These therapies are evidence-based and have the potential to improve both mental and physical health, leading to improved health outcomes for people with chronic illness and MHDs (Ee et al. 2020). The main aim of our review is to determine the effectiveness of lifestyle interventions in the management of patients with comorbid physical and MHDs in order to guide management, decrease burden of disease and improve QoL for these patients.

An initial search of PROSPERO and the *JBI Database of Systematic Reviews and Implementation Reports* was conducted. Two reviews with similar protocols were identified on PROSPERO (Registration numbers: CRD42019148544 and CRD42018096514). These reviews, however, only focus on randomised controlled trials (RCTs) or include only youth and adolescents. Our review will also consider a broader range of interventions than previously reported. These include behaviour change techniques and stress management modalities which were not included in the previous reviews. Previous systematic reviews and RCTs often focus on either the effects of lifestyle interventions on MHDs alone or on the chronic illness. However, many patients present with comorbidities, and this protocol addresses the need to investigate the effects lifestyle interventions may have on MHDs with comorbid chronic physical health disorders.

## Aim

The aim of our review will be to determine the effectiveness of lifestyle interventions on health-related QoL in the management of adults with comorbid mental and physical health disorders.

## Research question

The question our review aims to answer is: What is the effectiveness of lifestyle interventions compared with treatment as usual (or no treatment) to improve health-related QoL in adults with comorbid mental (depression, anxiety, bipolar disorder) and physical health disorder (cardiovascular disease, respiratory disorders, diabetes, obesity, metabolic syndrome and chronic pain)?

## Method

Our systematic review will be conducted using the Joanna Briggs’s Institute (JBI) methodology for systematic reviews of effectiveness (Tufanaru et al. 2020). It will be reported using the *Preferred Reporting Items for Systematic Reviews and Meta-Analysis* (PRISMA) statement (Page et al. 2021). This protocol has been registered on the Prospective Register of Systematic Reviews (PROSPERO). Registration number: CRD42021265704.

### Eligibility criteria

The review will include all experimental and quasi-experimental study designs. Only studies in English, published from 2011 to December 2022 will be considered for inclusion.

The Participants, Intervention, Comparator, Outcomes (PICO) framework will be used to answer the review question and to determine the exclusion and inclusion criteria. For our review, the population will include adults with comorbid mental (depression, anxiety, bipolar disorder) and chronic illness (cardiovascular disease, respiratory disorders, diabetes, obesity, metabolic syndrome and chronic musculoskeletal pain). The interventions will include the following lifestyle interventions: movement (physical activity and exercise), sleep hygiene, diet, stress management and cessation of substance use. Studies will be compared with those with no treatment or treatment as usual, and the outcomes will include health-related QoL (outcome measures may include the Short Form Health Survey: SF-36 and SF-12, the Sickness Impact Profile, WHO Quality of life [WHOQOL] Quality of Well-being Scale, EuroQOL-5 Dimension questionnaire [EQ-5D]). Studies of all methodological quality will be included provided they meet the inclusion criteria.

The following conditions will be excluded: schizophrenia, psychosis, pregnancy and pregnancy-related conditions (e.g. gestational diabetes and pre-eclampsia), coronavirus disease 2019 (COVID-19), organ transplants, acute hospitalisation (e.g. surgery or acute coronary syndrome) and neurological conditions (e.g. stroke, dementia and cognitive decline) and human immunodeficiency virus infection and acquired immunodeficiency syndrome (HIV and AIDS). Only interventions aimed at the individual patient will be considered.

### Search strategy

The JBI three-step search strategy will be used to identify studies, which will be included in the systematic review. This three-step search strategy started with developing a full strategy using the text words contained in the titles and abstracts of relevant articles identified during an initial literature search, and the Index or Medical Subject Headings (MeSH), identified during the initial search, used to describe the articles. The search strategy will be adapted for each specific database or information source. Step two involves searching each identified database for relevant studies. In step three, the reference list of included studies will be screened for possible inclusion in the full text screening. Authors of studies may be contacted if additional information is required (Tufanaru et al. 2020).

A librarian helped to develop the initial pilot search strategy and will help to adapt the search strategy to each database identified. The following databases will be searched: Medline (Ovid), CINAHL (EBSCO), SCOPUS, PEDRO, Cochrane Central Register of Controlled Trials as well as LiLACS. A preliminary search of MEDLINE (Ovid) was conducted to determine availability and suitability of articles that comply with the eligibility criteria (Appendix 1).

### Selection process

A pilot search will be conducted to ensure that the search strategy is appropriate and relevant studies are identified. All citations will be uploaded to EndNote (version 20) and duplicates will be removed. The JBI System for the Unified Management, Assessment and Review of Information (JBI SUMARI) software will be used to conduct the systematic review (Munn et al. 2019). The relevant citations and full-text versions of the articles will then be imported into the JBI SUMARI software program. All studies will be screened for inclusion by reviewing titles and abstracts initially and then reviewing of full text-versions in order to ensure they meet the inclusion criteria. The screening will be performed by two independent reviewers and disagreements will be discussed and if consensus is not achieved, a third reviewer will decide.

### Assessment for methodological quality

Following the screening process, the JBI critical appraisal tools (JBI CATs) will be used to assess the methodological quality of the included studies. The JBI CATs are specific to a specific study type, for example, JBI CAT for RCTs will be used to assess RCTs. Studies of all methodological quality will be included in the systematic review provided they meet the inclusion criteria.

### Data extraction

Two reviewers will extract the data independently. Any discrepancies will be settled by discussion and if the need arises, a third reviewer will make the final decision.

Data to be extracted will include participants, study methods, interventions, and outcomes related to the aim of the review and will be presented in a table. The main outcome will include health-related QoL.

### Data analysis

Using JBI SUMARI, if feasible, studies will be pooled, and a meta-analysis will be performed. If a meta-analysis is not possible, the findings will be presented in a narrative form and using tables and figures where it is suitable. The random effects model will be used for statistical analysis.

Odds ratios or weighted final post-intervention mean differences will be used to express the effect sizes, and a 95% confidence interval will be determined. To test for heterogeneity, chi-square and I-square tests will be applied. A funnel plot will be used in order to determine publication bias, if 10 or more publications are included in a meta-analysis (Tufanaru et al. 2015). A funnel plot asymmetry test will be carried out where applicable (Tufanaru et al. 2015).

### Assessing the certainty in the findings

The Grading of Recommendations, Assessment, Development and Evaluation (GRADE) approach for grading the certainty of evidence will be followed (Alonso-Coello et al. 2016; Guyatt et al. 2008) and a Summary of Findings (SoF) will be created using the GRADEPro Guideline Development Tool (McMaster University 2020). The SoF will present the following information where appropriate: absolute risks for the treatment and control, estimates of relative risk and will rank the quality of the evidence based on the direct risk of bias, precision and publication bias and heterogeneity of the review results (Tufanaru et al. 2020). The outcomes reported in the SoF will be: health-related QoL (Tufanaru et al. 2020).

### Ethical considerations

An ethical waiver was obtained from the University of the Witwatersrand Human Research and Ethics Committee (Medical) (Reference no.: W-CBP-220328-01), as the study is a review of information in the public domain and no human participants are included in our review.

## Discussion and conclusion

Mental health disorders are increasing globally, and many patients with MHDs have comorbid physical health disorders. Pain, chronic illness and psychiatric comorbidities all increase the burden of disease experienced by these patients and lead to a poorer QoL. As such, healthcare approaches are needed that have an effect on both physical and mental health concurrently (Ee et al. 2020). Previous research suggests that lifestyle interventions are effective management strategies for MHDs and NCDs and, as such, should form part of the treatment plan for patients to improve treatment outcomes and QoL of these patients.

Previous studies have focussed on lifestyle interventions for MHDs and chronic illness as separate conditions in separate studies, but as patients often present with multi-morbidity, more information is needed regarding the outcomes of lifestyle interventions in patients with comorbid physical and MHDs. Evidence suggests that lifestyle interventions have a positive effect on both MHDs and chronic illness and therefore may improve QoL. Our review therefore aims to determine the effectiveness of lifestyle interventions in order to develop evidence-based recommendations for healthcare professionals.

## Appendix 1: Search strategy for MEDLINE (Ovid)

Search for: limit 22 to (yr=”2011 - 2022” and “adult (19 to 44 years)” and english)

Results: 231

Database: Ovid MEDLINE(R) ALL <1946 to February 14, 2023>Search Strategy:

-----------------------------------------------------------------------------------

1. exp Bipolar Disorder/ (44691)
2. exp Depression/ (147312)
3. exp Anxiety/ or exp Anxiety Disorders/ (184353)
4. exp Cardiovascular Diseases/ (2683766)
5. exp Respiratory Tract Diseases/ (1686804)
6. exp Metabolic Syndrome/ (37607)
7. exp Diabetes Mellitus/ (497921)
8. exp Musculoskeletal Pain/ (7197)
9. exp Obesity/ (254958)
10. 1 or 2 or 3 (329566)
11. 4 or 5 or 6 or 7 or 8 or 9 (4726226)
12. exp Exercise/ (241601)
13. exp Diet/ (323841)
14. exp Stress, Psychological/ (151021)
15. exp Behavior Therapy/ (87793)
16. 12 or 13 or 14 or 15 (770270)
17. 10 and 11 and 16 (5964)
18. exp “Quality of Life”/ (259705)
19. exp Social Integration/ (264)
20. exp Community Participation/ (47535)
21. 18 or 19 or 20 (305632)
22. 17 and 21 (759)
23. limit 22 to (yr=”2011 - 2022” and “adult (19 to 44 years)” and english) (231)
